# A Reductive Metabolic Switch Protects Infants with Transposition of Great Arteries Undergoing Atrial Septostomy against Oxidative Stress

**DOI:** 10.3390/antiox10101502

**Published:** 2021-09-22

**Authors:** José David Piñeiro-Ramos, Otto Rahkonen, Virpi Korpioja, Guillermo Quintás, Jaana Pihkala, Olli Pitkänen-Argillander, Paula Rautiainen, Sture Andersson, Julia Kuligowski, Máximo Vento

**Affiliations:** 1 Neonatal Research Unit, Health Research Institute Hospital La Fe, Avenida Fernando Abril Martorell 106, 46026 Valencia, Spain; josedavidpineiro@gmail.com; 2 Department of Paediatric Cardiology, New Children’s Hospital, University of Helsinki and Helsinki University Hospital, Box 347, Stenbäckinkatu 9, 00029, Helsinki, HUS, Finland; Otto.Rahkonen@hus.fi (O.R.); jaana.pihkala@hus.fi (J.P.); Olli.Pitkanen@hus.fi (O.P.-A.); 3 Department of Children and Adolescents, Oulu University Hospital, P.O. Box 23, FIN-90029 OYS, 90570 Oulu, Finland; korpiojavirpi@gmail.com; 4 Health & Biomedicine Unit, Leitat Technological Center, Par Cientific Barcelona, 08028 Barcelona, Spain; gquintas@leitat.org; 5 Analytical Unit, Health Research Institute La Fe, Avenida, Fernando Abril Martorell 106, 46026 Valencia, Spain; 6 Department of Anaesthesia and Intensive Care, New Children’s Hospital, Helsinki University Hospital and University of Helsinki, Stenbackinkatu 9, 00029 Helsinki, Finland; Paula.Rautiainen@hus.fi; 7 Pediatric Research Center, New Children’s Hospital, Helsinki University Hospital and University of Helsinki, Stenbackinkatu 9, 00029 Helsinki, Finland; sture.andersson@hus.fi; 8 Division of Neonatology, University & Polytechnic Hospital La Fe, Avenida Fernando Abril Martorell 106, 46026 Valencia, Spain

**Keywords:** transposition of the great arteries, balloon atrial septostomy, hypoxemia, metabolomics, oxidative stress, newborn, liquid chromatography-mass spectrometry (LC-MS)

## Abstract

Transposition of the great arteries (TGA) is one of the most common cyanotic congenital heart diseases requiring neonatal surgical intervention. Parallel circulations that result in impaired cerebral oxygen delivery already in utero may lead to brain damage and long-term neurodevelopmental delay. Balloon atrial septostomy (BAS) is often employed to mix deoxygenated and oxygenated blood at the atrial level. However, BAS causes a sudden increase in arterial blood oxygenation and oxidative stress. We studied changes in oxygen saturation as well as metabolic profiles of plasma samples from nine newborn infants suffering from TGA before and until 48 h after undergoing BAS. The plasma metabolome clearly changed over time and alterations of four metabolic pathways, including the pentose phosphate pathway, were linked to changes in the cerebral tissue oxygen extraction. In contrast, no changes in levels of lipid peroxidation biomarkers over time were observed. These observations suggest that metabolic adaptations buffer the free radical burst triggered by re-oxygenation, thereby avoiding structural damage at the macromolecular level. This study enhances our understanding of the complex response of infants with TGA to changes in oxygenation induced by BAS.

## 1. Introduction

Transposition of the great arteries (TGA) is one of the most common cyanotic congenital heart diseases with an incidence of 0.3 per 1000 live births that requires surgical intervention in the neonatal period [1]. In hearts with TGA, systemic and pulmonary circulations run in parallel rather than in serial. This results in significant hypoxemia clinically reflected as central cyanosis. Survival after birth is only possible if there is an adequate blood mixing between the two circulations. Most hypoxemic neonates with TGA benefit from early institution of prostaglandin E1 (PGE) for ductal patency. If hypoxemia persists despite prostaglandin E1 (PGE1) infusion, balloon atrial septostomy (BAS) is needed to increase systemic oxygenation by improving the mixing of deoxygenated and oxygenated blood at the atrial level. After stabilization, arterial switch operation (ASO) is performed in the neonatal period. Mortality of ASO is low in the current era, however, morbidity is high and neonates with TGA are at risk of impaired neurodevelopmental outcome. Thus, long-term follow-up demonstrates that 30–50% of school-aged children with TGA show some form of developmental delay [2].

The underlying mechanism of developmental delay is thought to be multifactorial and include prenatal and postnatal factors. Hence, fetal hypoxia due to decreased oxygen delivery has been implicated in the abnormal brain development seen in newborns with TGA [3]. Moreover, reduced fetal cerebral oxygen consumption in TGA neonates has been associated with smaller head circumference and brain volume than those of normal neonates [4]. In addition, postnatal factors such as postnatal chronic hypoxemia, open-heart surgery with deep hypothermic circulatory arrest, and balloon atrial septostomy (BAS) have also been considered responsible for brain injury [5]. BAS improves mixing of systemic and pulmonary circulation and leads to an immediate increase in arterial oxygen content. However, BAS does not allow for full normalization of systemic oxygenation preoperatively. Very little is known about the direct effect of BAS on the neonatal brain, on cerebral oxygenation and oxygen metabolism [6], and whether the rapid increase of oxygen delivery results in brain reperfusion injury in neonates with TGA [7,8].

In mammals, aerobic metabolism with the concourse of oxygen is the most efficient biological means to supply energy required to sustain life. Under anaerobic conditions, pyruvate is converted into L-lactate. Anaerobic metabolism is by far less energy efficient than aerobic metabolism. Hence, in the absence of oxygen, the energy consumed by neurons rapidly leads to an exhaustion of the ATP reserves [9]. Due to the high metabolic rate of the brain, survival is almost exclusively dependent on the energy generated by aerobic glycolysis. The lack of oxygen stores and the reduced glycolytic capacity compel brain tissue to rely entirely on a continuous supply of oxygen and glucose provided by cerebral perfusion. Under these circumstances, acutely or chronically reduced oxygen availability due to environmental or pathophysiological causes inevitably leads to alterations of the brain structure and function [10].

Incomplete reduction of oxygen leads to the formation of reactive oxygen species (ROS), some of which are free radicals (e.g., anion superoxide and hydroxyl radicals). These extremely short half-life metabolites are capable of damaging nearby cellular components such as proteins, lipids, carbohydrates or DNA [11]. Both, acute and chronic hypoxia, enhance the formation of ROS through mitochondrial uncoupling provoking oxidative stress (OS) [12]. In addition, during reoxygenation, the increased availability of oxygen causes the activation of oxidases such as nicotinamide adenine dinucleotide phosphate (NADPH) oxidase or xanthine oxidase, further increasing the formation of anion superoxide and nitric oxide [11]. Neurons are highly vulnerable to the deleterious effects of ROS generated during acute hypoxia and/or hyperoxia. ROS trigger specific pathways that lead to apoptosis, necrosis, and inflammation of vulnerable areas of the brain causing long term neurodevelopmental, motor, and cognitive impairment [10].

Blood lactate has been largely employed as a surrogate for tissue hypoxia and/or ischemia. However, exclusive monitoring of serum lactate has neither provided sufficient insight into the magnitude of brain hypoxia nor conferred reliable prognostic information regarding long-term neurodevelopmental impairment [13,14]. More recently, comprehensive metabolic fingerprinting characterized by the simultaneous measurement of hundreds of metabolites from biological matrices has been increasingly employed for identifying predictive biomarkers or patient stratification [15].

In the present study we focused on the metabolic switch in infants with TGA after BAS. We performed serial analysis of lipid peroxidation byproducts as well as the plasma metabolome before and after BAS. This allowed us to study the impact of the rapid change in arterial blood oxygen content switching from a chronic hypoxic environment to an almost normoxic one, thus giving an insight into the dynamic hypoxia-related changes on the phenotypic level.

## 2. Materials and Methods

### 2.1. Study Population

We performed a prospective single center study to evaluate changes in cerebral oxygenation and metabolism before and for a period of 96 h following BAS in neonates with TGA. All patients with TGA admitted to Children’s Hospital, Helsinki University Hospital, between 1 January 2015, and 1 June 2017, were considered for inclusion in the present study. Inclusion criteria included term gestational age and simple TGA without any significant associated heart defects (i.e., patients with ventricular septal defect were excluded). Reasons for failure to enroll included unavailability of parents for the consent process or parental refusal.

The study protocol was approved by the Ethics Committee of Helsinki University Hospital. All procedures were performed in accordance with relevant guidelines and regulations and written permission by signing an informed consent form or phone permission in urgent cases was obtained from legal representatives.

Data collected included peripheral oxygen saturation (SpO_2_), mixed venous saturation, regional cerebral tissue oxygen saturation (rcSO_2_) measured by near infrared spectroscopy (NIRS), heart rate, blood pressure, blood lactate levels, pH, base excess, and hemoglobin prior to and following BAS. Blood samples for metabolic analysis were collected from arterial cannula 5 min prior to and 5 min, 6 h, 24 h, 48 h, 72 h, and 96 h following BAS. At each timepoint, 1 mL of blood was collected into lithium heparin tubes, centrifuged, aliquoted, and stored at −70 °C. Differences during rcSO_2_, preductal peripheral oxygen saturation, fractional tissue oxygen extraction (FTOE), and cerebral oxygen extraction (CEO_2_) were analyzed prior to and following BAS. Cerebral oxygen extraction was estimated from the difference of SaO_2_ and ScO_2_ as ScO_2_ is close to venous SO_2_. FTOE was calculated as CEO_2_/SaO_2_. Information regarding medications used prior to and following BAS was collected from electronic patient records.

### 2.2. Analytical Procedures

#### 2.2.1. Lipid Peroxidation Biomarkers

Biomarkers of lipid peroxidation were analyzed in 63 plasma samples following previously published procedures [16,17]. Deuterated internal standards (IS) (PGF_2__α_-d4 and 15-F_2t_-Isoprostane-d4) were purchased from Cayman Chemical Company (Ann Arbor, MI, USA). For sample processing, 100 μL of plasma were thawed on ice and 100 μL of KOH solution at 15% (*w/v*) were added. The mixture was incubated at 40 °C for 30 min. A volume of 3 μL of aqueous IS solution (20 μM) was added to hydrolyzed samples and diluted to 900 μL with H_2_O:MeOH (85:15, 2.8% *v/v* HCOOH) solution. Then, the samples were mixed for 30 s at maximum speed and centrifuged at 16,000× *g* and 4 °C for 10 min. For clean-up and pre-concentration of the samples, an SPE procedure employing Discovery^®^ DSC-18 SPE 96-well plates from Sigma Aldrich Química S.A (Madrid, Spain) was carried out. First, the stationary phase was equilibrated with 1 mL of MeOH and 1 mL of water. Then, the supernatant of the centrifuged and diluted sample was loaded followed by washing with 1 mL of H_2_O (0.1% *v/v* HCOOH, pH 3) and 500 mL heptane. Finally, cartridges were dried with room air and the compounds of interest were eluted with 4 × 100 μL ethyl acetate. The eluate was evaporated using a miVac centrifugal vacuum concentrator (Genevac LTD, Ipswich, UK) and dissolved in 60 μL H_2_O (0.1% *v/v* HCOOH, pH 3):CH_3_OH (85:15 *v/v*).

An Acquity-Xevo TQS system from Waters (Milford, MA, USA) operating in negative electrospray ionization (ESI^−^) mode was employed for UPLC-MS/MS analysis. A Waters BEH C_18_ column (2.1 mm × 100 mm, 1.7 μm, Waters, Wexford, Ireland) was used. Flow rate, column temperature, and injection volume were set at 450 μL min^−1^, 45 °C, and 9 μL, respectively. A binary mobile phase H_2_O (0.1% *v/v* HCOOH):CH_3_CN (0.1% *v/v* HCOOH) gradient with a total runtime of 7.0 min was run as follows: from 0.0 to 0.1 min 15% *v/v* CH_3_CN (0.1% *v/v* HCOOH) (mobile phase channel B); from 0.1 to 5.0 min % B increased up to 40%; from 5.0 to 6.0 min % B increased up to 75%; between 6.0 and 6.15 conditions were held constant at 75% B followed by the return to initial conditions (i.e., 15% B) between 6.15 and 6.25 min; conditions were maintained for 0.75 min for system re-equilibration. ESI interface conditions were selected as follows: capillary voltage was set to 2.9 kW; source and desolvation temperatures were 150 °C and 395 °C, respectively; and nitrogen cone and desolvation gas flows were 150 and 800 L h ^−1^, respectively. Parameters selected for determination of lipid peroxidation biomarkers are shown in Table 1.

#### 2.2.2. Untargeted Ultra-Performance Liquid Chromatography Coupled to Time-of-Flight Mass Spectrometry (UPLC-TOFMS) Metabolomic Analysis

Plasma samples were thawed on ice and homogenized on a Vortex mixer. 75 µL of cold acetonitrile were added to 25 µL of plasma, homogenized and kept on ice during 15 min followed by centrifugation at 16,000× *g* during 15 min at 4 °C. 80 μL of supernatant were collected and transferred to a 96 well plate, evaporated to dryness on a miVac centrifugal vacuum concentrator (Genevac LTD, Ipswich, UK) at room temperature and dissolved in 60 µL of an internal standard (IS) solution containing betaine-D_11_, methionine-D_3_, hypoxanthine-D_3_, cystine-D_4_, tyrosine-D_2_, prostaglandinF_2α_-D_4_, uridine-C^13^N^15^, reserpine, phenylalanine-D_5_, leucine enkephalin, caffeine-D_9_, and tryptophane-D_5_ with purities ≥ 99% at a concentration of 1.5 µM in H_2_O:CH_3_CN (0.1% HCOOH) (95:5 *v/v*).

A QC sample was prepared by mixing 5 μL of each plasma sample and a total of three aliquots were processed alongside with the plasma samples applying the same procedures. A blank extract was prepared by using a heparinized syringe and 0.5 mL of ultrapure H_2_O and processed as described for plasma samples.

For chromatographic separations, an Agilent Technologies (Santa Clara, CA, USA) 1290 Infinity UPLC chromatograph equipped with a UPLC ACQUITY BEH C18 column (2.1 mm × 100 mm, 1.7 μm, Waters, Wexford, Ireland) was employed. Autosampler and column temperatures were set to 4 and 40 °C, respectively. A flow rate of 400 μL min^−1^ and an injection volume of 4 μL were used. Separations were carried out keeping 98% of mobile phase A (H_2_O, 0.1% *v/v* HCOOH) for 0.5 min, followed by a linear gradient from 2 to 20% of mobile phase B (CH_3_CN, 0.1% *v/v* HCOOH) in 3.5 min and from 20 to 95% B in 4 min. Conditions of 95% B were maintained for 1 min and a 0.25 min gradient was used to return to the initial conditions, which were held until reaching 8.5 min.

Full-scan MS data were acquired between 100 and 1700 *m/z* with a scan frequency of 6 Hz (1274 transients/spectrum) on an iFunnel quadrupole time-of-flight (QTOF) Agilent 6550 spectrometer operating in the TOF MS mode. The following electrospray ionization settings were used: gas T, 200 °C; drying gas, 14 L min^−1^; nebulizer, 37 psig; sheath gas T, 350 °C; sheath gas flow, 11 L min^−1^. A mass reference standard used for automatic MS spectra re-calibration during analysis was introduced into the source via a reference sprayer valve using the 149.02332 (background contaminant), 121.050873 (purine), and 922.009798 (HP-0921) *m/z*. MassHunter workstation from Agilent was employed for data acquisition and manual integration of ISs.

Before launching the analytical sequence, system suitability was checked employing a standard mixture containing ISs. The analytical system was conditioned by eight repeated injections of the QC at the beginning of the batch. Data acquired during system conditioning were discarded from data analysis. A total of 63 plasma sample extracts were analyzed in randomized order in a single analytical batch using the positive electrospray (ESI^+^) mode. QC samples were analyzed every 6th sample and at the beginning and end of the batch for assessment and correction of instrumental performance [18]. The blank extract was injected a total of two times (once during system conditioning and once at the end of the batch) and used for data clean-up with the aim of identifying signals from other than biological origin. Subsequently, sample analysis was carried out in ESI^−^ mode repeating the same protocol described for the ESI^+^ mode.

#### 2.2.3. Data Processing and Statistical Analysis

ProteoWizard [19] (http://proteowizard.sourceforge.net (accessed on 20 September 2021)) software was used for conversion of raw UPLC-TOFMS data into centroid mzXML format. A peak table was extracted using XCMS (version 3.4.2) [20,21,22] (https://bioconductor.org/packages/release/bioc/html/xcms.html (accessed on 20 September 2021)) running in R (version 3.5). For peak detection, the centWave method was used as follows: ppm = 20, peakwidth = (4 and 25), snthresh = 10. For the resolution of overlapping peaks, a minimum *m/z* difference of 7.5 mDa was selected. For each extracted feature, the ‘wMean’ function was used for calculating intensity-weighted *m/z* values and peak limits used for integration were found through descent on the Mexican hat filtered data. For peak grouping the “nearest” method with mzVsRT = 1 and retention time (RT) and *m/z* tolerances of 6 s and 10 mDa, respectively, was used. Missing peak data was filled applying the fillPeaks method with the default parameters. A total of 18,582 and 13,479 features were initially detected after peak detection, integration, chromatographic deconvolution, and alignment in ESI^+^ and ESI^−^ modes, respectively. The CAMERA [23] package was used for identifying peak groups and annotation of isotopes and adducts using the following settings: sigma = 6, perfwhm = 0.5, ppm = 20.

Peak integration accuracy was checked by comparing the generated peak table with areas obtained from manual integration of ISs. Peak intensities of ISs and QC samples were used for assessing the instrumental response during data acquisition throughout the batch as described elsewhere [24,25]. The Quality Control-Support Vector Regression (QC-SVR) algorithm [26] and the LIBSVM library [27] were used for correcting intra-batch variation using an ε–range of 2.5 to 7.5 and a γ-range of 1 to 10^5^. C was defined for each feature as the median value in QCs. Then, features detected in blanks (<5× signal of the blank) and those with an RSD% in QC samples ≥20% were excluded. The final peak tables contained 3886 and 5600 features for ESI^+^ and ESI^−^, respectively, and were searched for molecular ion peaks of drugs and known drug metabolites that have been administered to infants, their isotope as well as Na and K adducts (*m/z* tolerance: 10 mDa). Metabolic features that were identified as drugs or their metabolites, isotopes and adducts were excluded from further data analysis.

MATLAB 2019b inbuilt functions as well as in-house written scripts (available from the authors upon reasonable request) and the PLS Toolbox 8.0 from Eigenvector Research Inc. (Wenatchee, WA, USA) were used for Principal Component Analysis (PCA) and the computation of Pearson correlations. For PCA, data sets generated in ESI^+^ and ESI^−^ were concatenated. MetaboAnalyst (version 4.0) [28] was used for hierarchical clustering and the generation of heatmaps employing Euclidean distance and Ward’s method (statistical analysis tool). Pathway analysis was carried out using MetaboAnalyst with the MS peaks to pathways tool (mass accuracy = 10 ppm, mummichog algorithm with top 10% peaks *p*-value cut-off) in the 4-column format (*m/z*, RT, ionization mode, *p*-value of Pearson correlation between metabolic features and FTOE) and the Kyoto Encyclopedia of Genes and Genomes (KEGG) pathway library (*Homo sapiens*). Metabolomics data are available on Zenodo (https://zenodo.org/record/4495124#.YBpwoC1DkWp (2 February 2021)).

The non-parametric Wilcoxon rank-sum test was used for assessing changes in levels of biomarkers of lipid peroxidation over time. *p*-values from Spearman correlations were used for clinical data, where appropriate.

## 3. Results

### 3.1. Clinical Results

**Figure 1:**
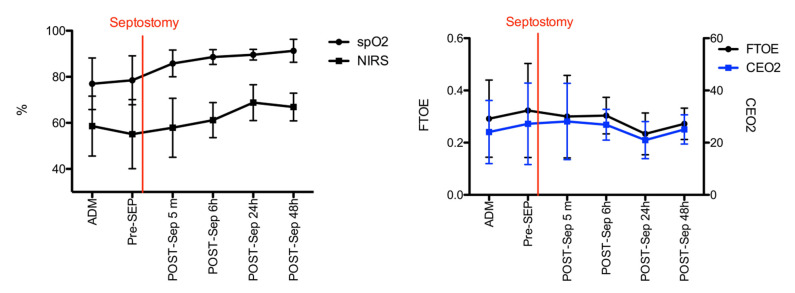
Evolution of SpO2, rcSO2 (**left**), FTOE, and CEO2 levels (**right**) before and after BAS. Black and blue lines and error bars are median and 25th and 75th percentile, respectively.

A total of 12 newborn infants fulfilled the study requirements. Out of these, one patient died, and two patients did not require BAS. The remaining 9 patients who underwent BAS, completed all analysis. Demographics and perinatal characteristics are detailed in Table 2. Six patients (50%) had prenatal diagnosis and nine patients (75%) underwent BAS due to low preductal saturation at the age 4.6 (± 2.7) hours. There was one early death prior to BAS. The patient was transferred from another central hospital with prostaglandin infusion but had extremely low preductal saturations (<30%), severe lactate acidosis, and was in pulseless electrical activity at the time of admission and care was withdrawn. Figure 1 describes evolving SpO_2_, rcSO_2_, FTOE, and CEO_2_ before and after BAS. In the nine patients included in the study, the lowest preductal peripheral oxygen saturation (SpO_2_) at admission had a median of 64.5% (range 39.0–92.0). Preductal SpO_2_ increased from a median of 85.6% (range 62.0–90.6) before BAS to 89.1% (range 81.8–93.5) 6 h following BAS and 90.0% (range 85.2–93.6) 24 h following BAS. rcSO_2_ at the same time points were 50.0% (range 35.0–70.0), 52.8% (range 36.4–72.5), 63.0% (range 48.2–74.1), and 69.2 (range 58.8–80.8), respectively. rcSO_2_ correlated strongly with simultaneously measured SpO_2_ (Spearman’s r = 0.89, *p*-value < 0.001). CEO_2_ increased after BAS (27.2–28.1) but both, CEO_2_ and FTOE, decreased 24 h following BAS (see Figure 1). Complete recovery of cerebral oxygen saturation did not occur until 24 h after BAS.

### 3.2. Lipid Peroxidation Biomarkers

**Figure 2:**
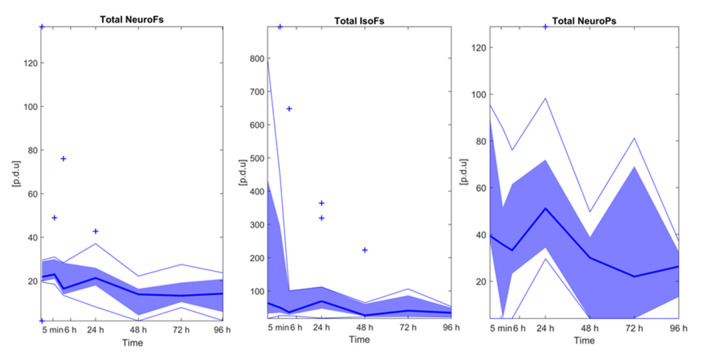
Isoprostanoid levels over time. Note: Bold blue lines correspond to median values, blue areas correspond to the interquartile range (1st and 3rd quartile), thin blue lines correspond to minimum and maximum values, and + correspond to outliers. Note: NeuroFs = neurofurans; IsoFs = isofurans; NeuroPs = neuroprostanes.

Total di-homo-isoprostanes, di-homo-isofurans, and isoprostanes were excluded as these parameters were found <LOQ in all study samples. Figure 2 depicts relative responses of total neurofurans, isofurans, and neuroprostanes obtained over time. No significative changes over time were detectable (Wilcoxon rank sum test, *p*-values > 0.05). Furthermore, no strong (Pearson correlation coefficients > |0.5|) and significant correlations were found between isoprostanoid levels and FTOE.

### 3.3. Effect of Time on the Plasma Metabolome

**Figure 3:**
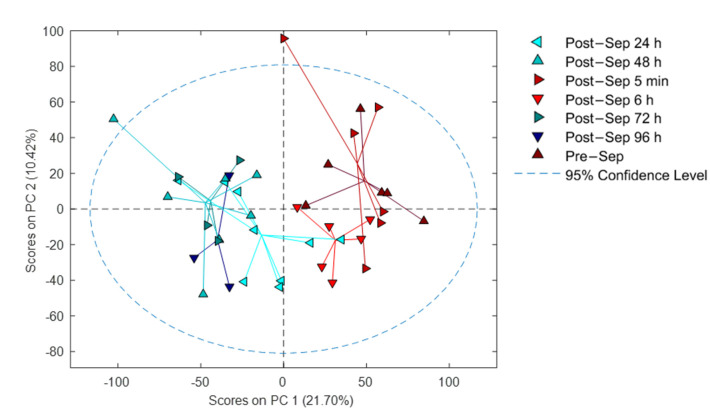
PCA results of samples collected before and at different time points after BAS. Note: Sep stands for septostomy.

Figure 3 shows a PCA scores plot of the plasma metabolic fingerprint of infants before BAS and at different time points after BAS. The scores plot from PC1 vs. PC2 illustrates the impact of the sampling time point on the plasma metabolome. For most patients, a time-dependent shift towards lower scores on PC1 with increasing time after septostomy was observed. Even though the applied data analysis workflow included the removal of drug metabolites, this effect might, at least partially, be related to the employed medication and the procedure itself. Also, a high inter-individual variation can be noted.

### 3.4. Correlation of Metabolic Features with FTOE

**Figure 4:**
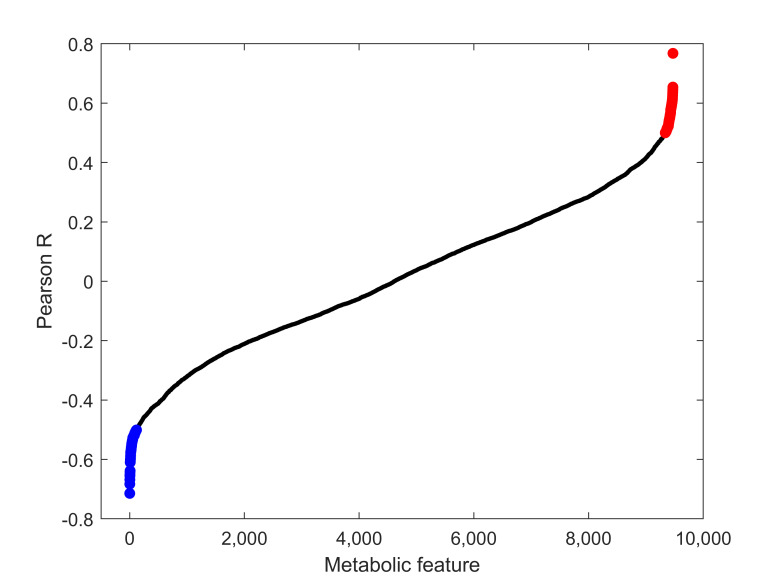
Pearson correlation coefficients between metabolic features and FTOE. Blue: features with *p*-values < 0.05 and correlation coefficients < −0.5; red: features with *p*-values < 0.05 and correlation coefficients > 0.5; black: all remaining features.

**Figure 5:**
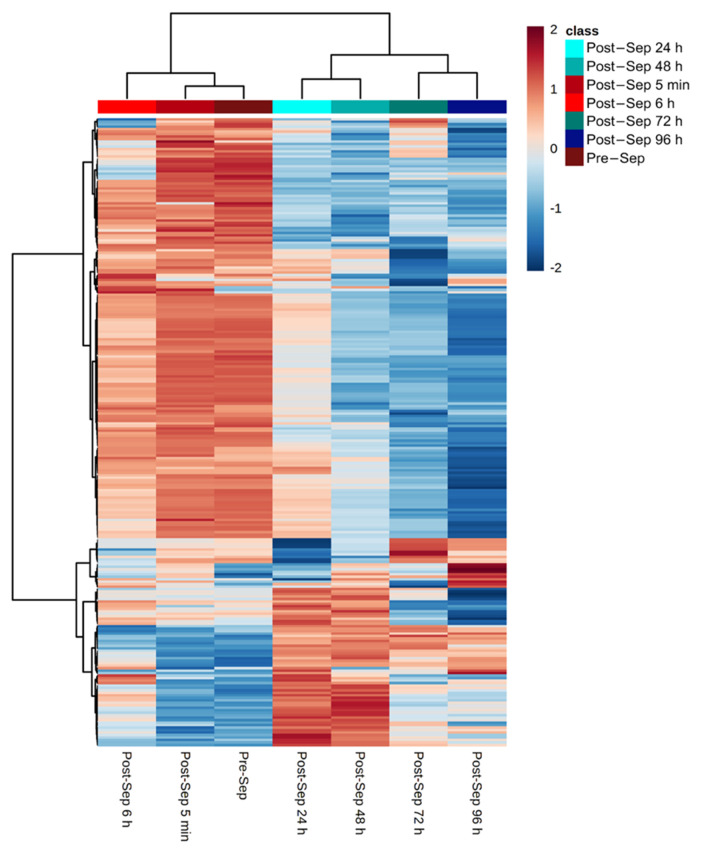
Relative intensities of metabolic features that correlate (i.e., *p*-value < 0.05 and |correlation coefficients| > 0.5) with FTOE. Note: color represents autoscaled relative intensities; blue—metabolite levels lower than average, and red—metabolite levels higher than average. *p*-values from Pearson correlation were computed and significantly correlating metabolites are shown; heatmap calculated using Euclidean distance and Ward algorithm.

FTOE reflects the balance between oxygen supply and consumption in tissue and can, therefore, be used as an indicator of inadequate tissue perfusion and oxygenation. We specifically focused on modelling the effects of metabolic changes in the plasma metabolome associated with FTOE. Pearson correlations for each metabolic feature with FTOE before and at different time points post-septostomy were calculated as shown in Figure 4. A panel of features with a significant association with FTOE (i.e., *p*-values from Pearson correlation < 0.05) was identified including both, positively (red dots, correlation coefficient > 0.5) and negatively (blue dots, correlation coefficient < −0.5) correlated features. Figure 5 shows a heatmap of the relative intensities of significantly correlated features. Two distinct clusters can be observed, with plasma fingerprints from samples collected before and 5 min as well as 6 h after BAS belonging to one cluster, and samples collected 24–96 h after BAS belonging to a second cluster. Most metabolic features in Figure 5 showed a decreasing trend in relative intensities when comparing cluster one to cluster two. Pathway analysis detected four significantly altered pathways associated with changing FTOE in infants with TGA undergoing BAS (see Table 3).

## 4. Discussion

TGA is a severe congenital cardiac malformation that causes hypoxemia during fetal life and in the newborn period [1]. TGA has deleterious consequences on growth and development due to a deficient tissue oxygenation that especially affects the Central Nervous System [2,4]. We report the first metabolomic study involving neonates with TGA who underwent BAS. We performed a study on oxidative stress biomarkers as well as a comprehensive qualitative characterization of plasma metabolites before and after BAS.

BAS in TGA patients caused a rapid switch from hypoxia to normoxia. Preductal peripheral pulse oximetry saturation and rcSCO_2_ increased rapidly after BAS while simultaneously FTOE decreased. As a result, brain oxygenation substantially improved (See Figure 1). Thereafter, changes in oxygenation plateaued. Complete recovery of cerebral oxygen saturation occurred only 24 h after BAS. A gradual change was observed in the metabolome, accordingly (See Figure 3). The provision of energy to satisfy metabolic demands of the brain is exclusively dependent on aerobic metabolism and oxygen deprivation caused by hypoxia and/or ischemia for just a few minutes may cause severe brain damage. Mitochondrial ROS production is regulated through tissue succinate levels and the activity of oxidases (NADPH oxidase, xanthine oxidase) [29]. During hypoxia and ulterior reoxygenation both, succinate levels and oxidase activation, generate a burst of ROS that directly damage tissue structure and function [29]. Moreover, the pro-oxidant imbalance provokes the activation of the caspase pathway and of transcription factor NF*k*B. Subsequently, programmed cell death and inflammation are triggered for hours, days, or even weeks. Consequently, there is an amplification of the initial area of brain injury that contributes to aggravate long-term neurological prognosis [30,31,32].

Isoprostanes and isofurans, but especially neuroprostanes and neurofurans are highly sensitive to oxidative stress related brain damage [33]. However, we did not observe changes in those compounds after BAS (See Figure 2). The attenuation of the expected pro-oxidant status after BAS suggests a pattern of metabolomic changes with a reducing profile. In this regard, post-BAS untargeted metabolomics evidenced a significant enhancement in the activity of the pentose phosphate pathway (PPP). The central role of the PPP has attracted more attention in recent years. Emerging evidence suggests that the PPP is tightly and meticulously controlled in cells and that its abnormal regulation leads to uncontrolled redox homeostasis [34]. The PPP has shown great versatility for *de novo* nucleotide biosynthesis via ribose-5-phosphate and adopts a simultaneous organization with glycolysis to produce NADPH. Nucleotide biosynthesis possibly participates in DNA damage repair. Our pathway analysis data revealed changes in the relative concentrations of 2-deoxy-D-ribose 1-phosphate (C00672), a precursor of ribose-5-phospate, and substrate of phosphoribosyl pyrophosphate (PRPP). This substrate is an essential compound of purine, pyridine, and histidine synthesis. NAPDH is a cofactor of glutathione reductase, an essential enzyme in the glutathione redox cycle that contributes to the reconversion of GSSG into GSH thus contributing to the normalization of the GSH/GSSG pair and the removal of ROS [35,36]. In this context, the pathway analysis data suggest an alteration of gluconic acid (C00257). The phosphorylation of this compound generates 6-phosphogluconate, an essential substrate in the oxidative branch of the PPP. Furthermore, alterations of other compounds such as ribitol-5-phosphate (C01068) and 2-deoxy-D-ribose (C00672) contribute as substrates for key PPP compounds. Indeed, the activity of the PPP is rapidly re-routed when cells are exposed to an oxidative burst. This response is exquisitely adjusted by cooperating of metabolic and gene regulatory mechanisms. Metabolic changes imply the inactivation of glycolytic enzymes which occurs immediately after the oxidant aggression thus blocking glycolysis [37]. Thereafter, the transcriptional response takes over and maintains higher PPP activity through up-regulation of enzymes and post-translational modifications including those which increase the activity of G6PDH [38]. Furthermore, to counteract mitochondrial ROS production under normal metabolic circumstances, but also during hypoxia or hypoxia-reoxygenation, steady NADPH production becomes essential as it represents the main electron donor for the generation of GSH that will provide electrons for the reduction of detrimental peroxides by glutathione peroxidase [34].

In addition, our results reveal the alteration of the pentose and glucuronate interconversion pathway, another key pathway in the homeostasis of metabolic pathways. The glucuronate pathway is an alternative pathway for the oxidative degradation of glucose without the production of ATP. Substrate compounds such as xylose (C00181), arabinose (C00259), arabitol (C00532), xylulose (C00312), UDP-glucose (C00029), glucuronic acid (C00191), dehydrogulonate (C00618), xylonolactone (C02266), and ribitol-5-phosphate (C01068) were altered according to the reported pathway analysis data. Interestingly, in humans the synthesis of ascorbic acid is not feasible, therefore a substantial proportion of uridine diphosphate glucoronate (UDP-glucoronate) is converted into xylulose-5-phosphate which is further metabolized through the PPP to fuel NADPH production and promote the preservation of a reduced environment [39].

Finally, pathway analysis also showed an alteration of ascorbate and aldarate and inositol phosphate metabolism. The ability of ascorbate to donate electrons enables it to act as a free radical scavenger and to reduce higher oxidation states of iron to Fe^2+^. Ascorbic acid is an important antioxidant in plasma, where it consumes oxygen free radicals. Erythrocytes have a high capacity to regenerate ascorbate from its two electron-oxidized form, dehydroascorbic acid. Intracellular dehydroascorbic acid is rapidly reduced to ascorbate by GSH in a direct chemical reaction, or indirectly with the concurrent action of glutaredoxin and thioredoxin reductases. Intracellular ascorbate can spare, and possibly recycle, alpha distocopherol in the erythrocyte membrane. In turn, alpha tocopherol protects the cell membrane from lipid peroxidation. The ability of erythrocytes to recycle ascorbate, coupled with the ability of ascorbate to protect alpha tocopherol in the cell membrane and in lipoproteins, provides a potentially important mechanism for preventing lipid peroxidative damage secondary to hypoxia or hypoxia-reoxygenation events [40].

We acknowledge limitations of our study. First, the number of subjects included is limited and some of the blood samples during BAS were not collected. We would like to stress the stringent inclusion criteria applied during patient recruitment and the low incidence of the condition. The present data provides evidence to justify large multi-center efforts for validating the current findings. Finally, we lack a control group of healthy infants for obvious ethical reasons.

## 5. Conclusions

In summary, this is a comprehensive metabolomic assessment of neonates with TGA. The results obtained suggest differences in oxygen supply and consumption in cerebral tissue during hypoxia and near-normoxia. The number of patients is limited, but the combined assessment of lipid peroxidation biomarkers and untargeted metabolomic screening of a cohort of infants with TGA undergoing BAS provides insightful information to understand the physiopathology of this complex disease. The metabolic switch after BAS causes oxidative stress. However, oxidative stress may at least be partially neutralized by the induction of different metabolic pathways but especially the PPP that supplies with reductive electrons. From a clinical point of view, although supplemental arterial oxygenation has limited effects on oxygenation in parallel circulation, our results suggest potential benefits of avoiding hyperoxia in patients undergoing BAS to prevent from attenuating the antioxidant effect inherent to the metabolic switch after septostomy.

## Figures and Tables

**Table 1:** Mass spectrometric parameters and chromatographic windows employed for the lipid peroxidation biomarkers.

Analyte [p.d.u.]	RT [min]	Parent Ion (*m/z*)	Daughter Ion (*m/z*)	CE (eV)	Cone Voltage (V)
IsoPs	4.3–6.6	353.20	115.00	30	35
Di-homo-IsoPs	5.0–6.8	381.00	143.00	20	20
Di-homo-IsoFs	3.5–6.5	397.00	155.00	24	35
NeuroFs	2.70–6.50	393.00	193.00	20	35
IsoFs	2.1–6.60	369.20	115.00	20	45
NeuroPs	2.30–6.50	377.00	101.00	20	35

Note: IsoPs = isoprostanes. IsoFs = isofurans. NeuroFs = neurofurans. NeuroPs = neuroprostanes. RT stands of Retention times. CE stands of collision energy.

**Table 2:** Patients’ demographics and timing of postnatal clinical and analytical interventions.

Patients’ Demographics	All Patients	BAS Patients
Patients recruited, N (%)	12 (100)	9 (100)
Gender, N (%)		
- Male	10 (83)	8 (89)
- Female	2 (17)	1 (11)
Balloon atrial septostomy, N (%)	9 (75)	9 (100)
Expired, N (%)	1 (8.3)	0 (0)
Prenatal diagnosis, N (%)	6 (50)	4 (44)
Gestational age, weeks (median, range)	39.5 (37.0–41.3)	39.9 (38.4–41.3)
Birth weight, kg (median, range)	3.4 (2.0–4.0)	3.5 (3.1–4.0)
Postnatal age, hours (median, range)		
- At the time of the first measured peripheral saturation	1.7 (0.5–13.2)	2.3 (0.5–6.7)
- At time of BAS		3.2 (2.0–9.1)
Saturation, % (median, range)		
- First peripheral saturation (SpO_2_)	79 (51–91)	77 (56–88)
- Lowest peripheral saturation (SpO_2_)	65 (39–86)	65 (39–86)
- First regional cerebral oxygen saturation (rcSO_2_)	58 (37–79)	53 (37–79)
- 5-min post septostomy peripheral saturation (SpO_2_)	86 (62–91)	86 (62–91)
Blood lactate, mmol/L (median, range)		
- Before BAS	3.2 (1.3–4.9)	3.6 (1.3–4.9)
- 5-min after BAS	2.7 (1.4–5.0)	3.3 (1.4–5.0)

**Table 3:** Pathway alterations associated with changing FTOE in infants with TGA undergoing atrial septostomy.

Pathway Name	Compound Code (KEGG)	Pathway ID	# Hits	# Sig Hits	*p*-Value
Pentose phosphate pathway	C01801; C00672; C00121; C00121; C00257; C00257; C00258	map00030	7	7	0.00002
Pentose and glucuronate interconversions	C01068; C00181; C00259; C00310; C00312; C00379; C00532; C00379; C00532; C00379; C00379; C00532; C00257; C00257; C00029; C00052; C00191; C00618; C02266	map00040	11	7	0.003
Ascorbate and aldarate metabolism	C00137; C00029; C02670; C00191; C00800	map00053	8	5	0.013
Inositol phosphate metabolism	C00137; C00222; C00191	map00562	4	3	0.03

Note: Mummichog input: 10 ppm; *p*-value cut-off: 10%; KEGG database. #: stands for number (Number of hits, number of significant hits).

## Data Availability

Metabolomics data are available on Zenodo (https://zenodo.org/record/4495124#.YBpwoC1DkWp (2 February 2021)).
